# Metabolomics Analysis on Mice With Depression Ameliorated by Acupoint Catgut Embedding

**DOI:** 10.3389/fpsyt.2021.703516

**Published:** 2021-08-03

**Authors:** Lining Duan, Wenhui Qiu, Guiqin Bai, Yiqi Qiao, Shiyu Su, Po-Chieh Lo, Yantong Lu, Guofeng Xu, Qi Wang, Min Li, Yousheng Mo

**Affiliations:** ^1^Science and Technology Innovation Center, Guangzhou University of Chinese Medicine, Guangzhou, China; ^2^Clinical Medical College of Acupuncture Moxibustion and Rehabilitation, Guangzhou University of Chinese Medicine, Guangzhou, China; ^3^Guangdong Provincial People's Hospital, Guangzhou, China; ^4^The Second Affiliated Hospital of Guangzhou University of Chinese Medicine, Guangzhou, China

**Keywords:** acupoint catgut embedding, depression, corticosterone, brain, metabolomics

## Abstract

Depression is a prevalent mental disease characterized by persistent low mood, lack of pleasure, and exhaustion. Acupoint catgut embedding (ACE) is a kind of modern acupuncture treatment, which has been widely used for the treatment of a variety of neuropsychiatric diseases. To investigate the effects and underlying mechanism of ACE on depression, in this study, we applied ACE treatment at the Baihui (GV20) and Dazhui (GV14) acupoints of corticosterone (CORT)-induced depression model mice. The results showed that ACE treatment significantly attenuated the behavioral deficits of depression model mice in the open field test (OFT), elevated-plus-maze test (EPMT), tail suspension test (TST), and forced swimming test (FST). Moreover, ACE treatment reduced the serum level of adreno-cortico-tropic-hormone (ACTH), enhanced the serum levels of 5-hydroxytryptamine (5-HT), and noradrenaline (NE). Furthermore, metabolomics analysis revealed that 23 differential metabolites in the brain of depression model mice were regulated by ACE treatment for its protective effect. These findings suggested that ACE treatment ameliorated depression-related manifestations in mice with depression through the attenuation of metabolic dysfunction in brain.

## Introduction

Depression is a prevalent mental disease characterized by persistent low mood, lack of pleasure and exhaustion (1). According to the World Health Organization, more than 350 million individuals suffer from depression worldwide (2). More seriously, depression will become the second leading cause of disability by the year 2030 (3). At present, a combination of pharmacotherapy and psychological treatment is the preferable strategy for depression management (4). Unfortunately, the expensive treatment costs and intolerable adverse side effects greatly limit its clinical efficacy (5). Therefore, it is urgent to develop a safer and more effective method to treat depression.

Although, many hypotheses have been suggested, the exact pathogenesis of depression is still not well-understood (6). Hypothalamic-pituitary-adrenal (HPA) axis is a primary neuroendocrine axis which regulates the stress response in mammals (7). Chronic psychosocial stress can damage the negative feedback system of HPA axis and increase cortisol levels in patients with depression (8). High levels of cortisol increase the adrenocorticotrophic hormone (ACTH) synthesis and adversely affect the hippocampus, which play a key role in the processing of emotional stimuli and regulation of stress responses (9, 10). The dysfunction of HPA axis is an important feature of the pathophysiology of depression (11). Furthermore, increasing evidence suggests that metabolic abnormalities play an important role in the pathogenesis of depression (12–14). A recent systematic review on biomarkers for major depressive and bipolar disorders showed that the metabolic pathways served key roles in the pathophysiology of depression and they predominantly centered on glutamatergic metabolism, energy metabolism and neurotransmission (15, 16). Moreover, a clinical study demonstrated that polyunsaturated fatty acids metabolism, purine metabolism and inosine could be the potential independent diagnostic biomarkers for major depressive disorder (17). These findings strongly indicated that metabolic abnormalities may be a new target for depression diagnosis and treatment. Metabolomics is a novel biological approach which can comprehensively and quantitatively isolate and characterize the metabolites, whose changes can be considered as the ultimate response of body to physiological and pathological changes (18, 19). It has significant potential in the discovery of biomarkers for disease and the elucidation of therapeutic mechanisms (20, 21).

Acupuncture is a widespread therapy for depression which has been proven (22). A meta analysis including 2,268 participants demonstrated that acupuncture showed clinically significant reductions in the severity of depression. Moderate-quality evidence from a systematic review of 21 trails demonstrated that the overall risk of adverse events is higher with fluoxetine (FLX) or paroxetine than with acupuncture (23). Acupoint catgut embedding (ACE) is a kind of modern acupuncture treatment. Catgut is a type of absorbable surgical suture. It could be embedded in specific acupoints and the acupoints are stimulated continuously for 7–14 days until the catgut is absorbed by the body (24, 25). ACE treatment overcomes the insufficiency of acupuncture treatment such as long treatment cycle, frequent frequency and short stimulation time, and it has been widely used for the treatment of a variety of neuropsychiatric diseases (26, 27).

To investigate the effects and underlying mechanism of ACE on depression, in this study, we applied ACE treatment at the Baihui (GV20) and Dazhui (GV14) acupoints of corticosterone (CORT)-induced depression model mice. Behavior examination was conducted to evaluate the effect of ACE on depressive-like behaviors. Enzyme linked immunosorbent assay (ELISA) was conducted to determine the serum levels of ACTH, 5-hydroxytryptamine (5-HT) and noradrenaline (NE). Liquid chromatography tandem mass spectrometry (LC-MS/MS)-based untargeted metabolomics analysis was performed to characterize the metabolic alterations in brain tissues. Our study provided new information on the effect and underlying mechanism of ACE in depression treatment.

## Materials and Methods

### Animal Groups

A total of 80 male Swiss mice (20–25 g, 7 weeks) were purchased from Guangdong Medical Laboratory Animal Center (Guangzhou, China). The mice were maintained with enough food and water at 24°C, 60% relative humidity and 12/12-h light/dark cycle. The mice were randomly divided into five groups (*n* = 16/group): control group (CON), CORT group (CORT), CORT with ACE treatment group (ACE), CORT with sham-ACE treatment group (SHAM), and CORT with fluoxetine (FLX) treatment group (FLX). FLX, a selective 5-HT reuptake inhibitor, is a first-line and widely used medication for depression. In this study, FLX was selected as a positive control to validate the efficacy of ACE treatment. CORT (Macklin, China) was dissolved in a saline solution containing 0.1% dimethyl sulfoxide and 0.3% Tween-80. To induce a depression model, the mice in the CORT, ACE, SHAM, and FLX groups received 20 mg/kg CORT suspension daily at 9:00 am through subcutaneous injections for 21 consecutive days as previously described (28). While the mice in the CON group received an equal volumes of saline containing 0.1% dimethylsulfoxide and 0.3% Tween-80.

### Treatment

**Figure 1:**
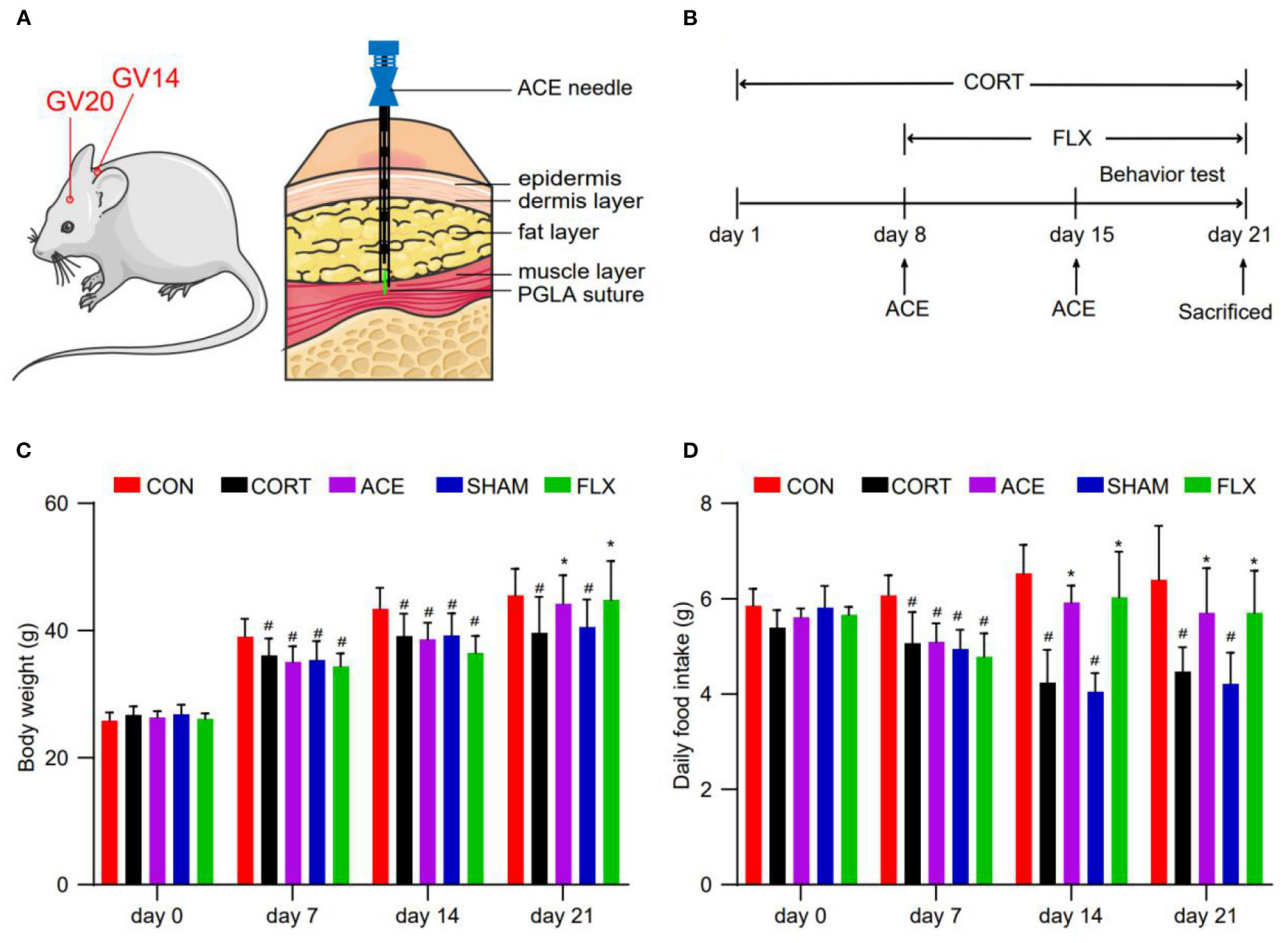
Effect of ACE on the body weight and daily food intake of mice. **(A)** ACE treatment demonstrating the position of acupoints (GV20 and GV14) in mice. **(B)** Experimental schedule of this study. **(C)** The body weight of mice in all subgroups were weighted every week during the experimental period at 8 a.m. (*n* = 16 per group). **(D)** The daily food intake of mice in all subgroups were weighted every week during the experimental period at 8 a.m (*n* = 16 per group). Experimental values were expressed as means ± SD. ^#^*P* < 0.05 compared with the CON group. *P < 0.05 compared with the CORT group.

All treatment measures were conducted 1 h later after CORT or saline administration. The mice in the ACE group were treated with ACE at GV20 and GV14 once on days 8 and 15. In mice, as in humans, the GV20 point is located at the intersection of the line linking the two mouse ear tips and the sagittal midline (29). The GV14 acupoint is located on the posterior midline and in the depression below the spinous process of the 7th cervical vertebra in the prone position (30). ACE needles 0.6 × 60 mm (Suzhou Medical Appliance) and Poly Lactic-co-Glycolic Acid (PLGA) absorbable sutures 0.2 × 3 mm (Suzhou Medical Appliance) were used for the implantation. GV20 and GV14 acupoints were sterilized with 70% alcohol solution and the sutures were embedded at the 5 mm depth of acupoints using the ACE needles (Figure 1A). While the mice in the SHAM group were administrated with sham ACE treatment once on days 8 and 15. Briefly, GV20 and GV14 acupoints were inserted with the ACE needles to a depth of 5 mm without sutures implantation. FLX (Macklin, China) was dissolved in 0.9% sterile saline. The mice in the FLX group were treated with 15 mg/kg FLX once a day through oral gavage for 14 consecutive days (day 8–21). The mice in the CON and CORT groups received same grasping and fixing manipulation (Figure 1B).

### Behavioral Tests

#### Body Weight and Daily Food Intake Measurement

In this study, the body weights and daily food intake of mice in each group were detected every week during the experimental period at 8 a.m.

#### Open Field Test

OFT was a well-established test to assess the locomotor activity, exploration habits and depression in the mice (31). OFT was performed in an open box structure (50 × 50 × 40 cm) with a black square at the bottom. The mouse was gently placed into the center of the square and allowed to move freely in the open field for 6 min. The entire test consisted of 1 min of acclimation and 5 min of test. The percentage time in the center and the proportion traveled in the center were recorded during the 5-min test period. After each mouse was tested, the open field was wiped and cleaned with 20% alcohol solution to eliminate smell interference.

#### Elevated-Plus-Maze Test

EPMT was used to evaluate the exploratory and anxiety-like behaviors in mice (32). The elevated-plus maze for mice consisted of two perpendicular open arms (35 × 5 cm) and two perpendicular closed arms (35 × 5 × 20 cm) connected by a central platform (5 × 5 cm), 50 cm above the floor. In this test, each mouse was placed at the center of maze and allowed to move freely in the maze for 6 min. Each mouse was allowed to acclimate for 1 min before the test was started. The number of open arm entries (OE), the number of close arm entries (CE), the time spent in the open arm (OT), and the time spent in the close arm (CT) were recorded in the 5-min test session. The percent of time spent in the open arm (OT%) = OT/(OT + CT) × 100%. The percent of entries in the open arm (OE%) = OE/(OE + CE) × 100%. The maze was cleaned with a solution of 20% ethanol solution between the sessions.

#### Tail Suspension Test

TST is a test of behavioral despair and is commonly used to assess depressive-like behavior (33). In the TST, each mouse was suspended individually by the tail on the edge of a shelf placed 50 cm above a table. The mouse was secured in the place *via* adhesive tape positioned ~0.5 cm from the tip of the tail. The duration of immobility was recorded for a test time of 5 min. A video camera positioned in front of the animal was utilized to track the movement of each animal. The videorecords were analyzed using a software (Super Maze, Shanghai Xinruan Information Technology Co, Ltd., Shanghai, China) to make the trajectory diagrams of mice in TST.

#### Forced Swimming Test

FST was conducted to evaluate the despair-like behavior of mice in each group (34). In the test, the mouse was gently placed into a clear glass cylinder (diameter 11 cm, height 30 cm) containing 25°C water to a depth of 20 cm. The duration of immobility was recorded during the last 5 min of the 6 min test session. Akin to TST, a video camera was placed in front of the animal to track the movement of each animal. The videorecords were analyzed by the software to make the trajectory diagrams of mice in FST.

### Serum Sample Preparation

After behavioral analyses, the eyeballs of mice in each group were removed and the blood was collected. Blood samples were stood at room temperature for 2 h and then they were centrifuged at 3,500 rpm for 15 min to separate serum. The serum samples were separated and stored in a −80°C freezer until analysis.

### ELISA Assay

The ELISA kits of ACTH, 5-HT and NE were obtained from Jiangsu Meimian company (Nanjing, China). Serum ACTH, 5-HT, and NE levels of mice in each group were detected using the ELISA kits according to the instructions. The optical density was measured at 450 nm using a microplate reader (MULTISKAN EX, Thermoscientific). Serum ACTH, 5-HT, and NE levels were calculated from the standard curve.

### Metabolomics Analysis

The metabolomics analysis work was completed by Shenzhen Huada Gene Technology Co., Ltd. (Shenzhen, China).

### Brain Tissue Samples Preparation

After the behavioral experiments were finished, six mice from the CON group, CORT group and ACE group were anesthetized and then transcardially perfused with PBS. Brains were quickly removed, flash frozen in liquid nitrogen and stored at −80°C. Before detection, 25 mg brain tissues were fixed with 800 μl extraction buffer (methanol:acetonitrile:water = 2:2:1, v:v:v, −20°C). The samples were placed in a tissue grinding machine and ground for 5 min at 50 HZ. Afterwards, the samples were centrifuged at 25,000 rcf for 15 min at 4°C, and then the supernatant was removed. Next, a total of 600 μl supernatant were dried with a vacuum concentration meter and added with reconstitution solution (methanol:water = 1:9, v:v). The samples were centrifuged again and the supernatant were placed in an autosampler vial for LC-MS/MS detection.

### LC-MS/MS Analysis

Waters 2D UPLC system (Waters Corp., Milford, MA, USA) and Q Exactive tandem mass spectrometer (Thermo Fisher Scientific, USA) were used for metabolite separation and detection. Chromatographic separation was conducted using a BEH C18 column (1.7 μm 2.1^*^100 mm, Waters, USA). The linear gradient programme set as follows: 2% B, 0–1 min; 2–98% B, 1–9 min; 98% B, 9–12 min; 98–2% B, 12.0–12.1 min. 2% B, 12.1–15 min. Flow rate was set 0.35 mL/min and the column temperature was 45°C. The injection volume was 5 μl.

MS data were collected with a Q Exactive tandem mass spectrometer (Thermo Fisher Scientific, USA). The mass spectra was a range of 70–1,050 mass-to-charge ratio with primary resolution of 70,000 and secondary resolution of 17,500. The ion source was electrospray ionization. The sheath gas flow rate was 40 and the aux gas flow rate was 10. The capillary temp was 320°C and the aux gas heater temp was 350°C.

### Data Analysis

The data were processed using Compound Discoverer 3.0 (Thermo Fisher Scientific, USA), including peak alignment, peak extraction and compound identification. Partial least squares method-discriminant analysis (PLS-DA) was constructed and variable importance in projection (VIP) scores were calculated to estimate the importance of each variable in the PLS-DA projection. Univariate analysis and student's *t*-test were used for statistical analysis to evaluate the significant difference of potential biomarkers.

### Kyoto Encyclopedia of Genes and Genomes Enrichment Analysis

The KEGG database was utilized to identify the relevant metabolic pathways and obtain a comprehensive metabolic bubble plot in the present work. In this study, KOBAS 2.0 software with the hyper-geometric test was used for KEGG pathway enrichment analysis. The differentially expressed metabolites file, the file including all the metabolites and the KEGG annotation file were input into the the software. The differentially expressed metabolites were set as foreground and all the metabolites were set as background. Functional enrichment analysis was estimated with the Fisher exact probability using the Gaussian hypergeometric test. KEGG pathways with a corrected *P*-value < 0.05 were considered significantly enriched. Finally, a bubble plot for significant metabolic pathway enrichment analysis was established using the software.

### Statistical Analysis

Experimental data were shown as mean ± standard deviation (SD), and they were analyzed by the Statistical Package for the Social Sciences software (SPSS; version 25.0). Comparison between two groups was performed using *t*-tests. Comparisons between multiple groups were conducted using one way analysis of variance (ANOVA) followed by the least significant difference (LSD) test. The *P*-value < 0.05 indicated statistical significance.

## Results

### Effect of ACE on the Body Weight and Daily Food Intake of CORT-Treated Mice

Before the experiment, no difference was observed in body weight among the groups. Compared with the control, CORT administration obviously inhibited the body weight gain in mice. Compared with the CORT group, ACE and FLX treatment significantly promoted body weight gain. While sham-ACE treatment could not reverse the inhibitory effect of CORT on body weight increase (Figure 1C). Furthermore, compared with the CORT and SHAM groups, ACE and FLX treatment remarkably enhanced the daily food intake of mice (Figure 1D).

### Effect of ACE on CORT-Induced Depression-Like Behavior in Mice

**Figure 2:**
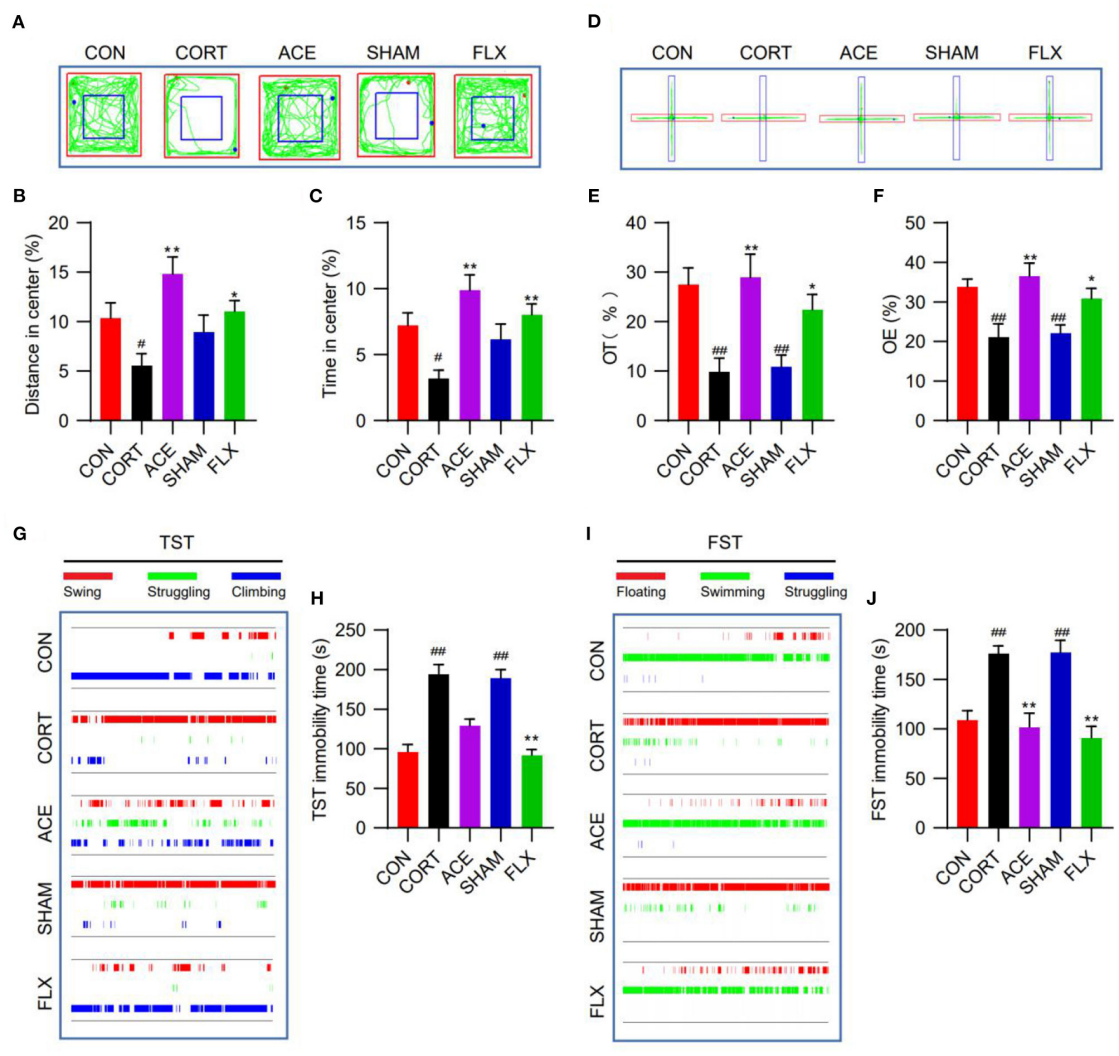
Effect of ACE on the depression-like behavioral deficits of mice. **(A)** The representative trajectory diagrams of mice in OFT. **(B)** ACE significantly increased the distance in center of mice in OFT (*n* = 16 per group). **(C)** ACE significantly increased the time in center of mice in OFT (*n* = 16 per group). **(D)** The representative trajectory diagrams of mice in EPMT. **(E)** ACE significantly increased the OT% of mice in EPMT (*n* = 16 per group). **(F)** ACE significantly increased the OE% of mice in EPMT (*n* = 16 per group). **(G)** The representative trajectory diagrams of mice in TST. **(H)** ACE decreased the immobility of mice in TST to some degree without statistical significance (*n* = 16 per group). **(I)** The representative trajectory diagrams of mice in FST. **(J)** ACE significantly decreased the immobility of mice in FST (*n* = 16 per group). Experimental values were expressed as means ± SD. ^#^*P* < 0.05 and ^##^*P* < 0.01 compared with the CON group. *P < 0.05 and **P < 0.01 compared with the CORT group.

OFT test showed that ACE and FLX treatment markedly improved the percent of time spent in the center and the percent of distance in the center in comparison with CORT group. While sham-ACE treatment showed no significant effect on the percent of time spent in the center and the percent of distance in the center (Figures 2A–C). In the EPMT, ACE, and FLX treatment significantly enhanced the OT% and OE% of mice in relative to the CORT group and SHAM group. These results indicated that the administration of CORT suppressed the locomotor activity and spontaneous exploratory behavior of mice, while ACE and FLX treatment effectively reversed these effects (Figures 2D–F). TST test revealed that CORT administration significantly increased the immobility time of mice compared with the control. While ACE could reduce the immobility time to some degree (Figures 2G,H). Similarly, ACE and FLX treatment effectively decreased the immobility time in FST in relative to the CORT and SHAM group (Figures 2I,J). These results indicated that ACE treatment could ameliorate the CORT-induced despair behavior in mice.

### Effect of ACE on Serum ACTH, 5-HT, and NE Levels

**Figure 3:**
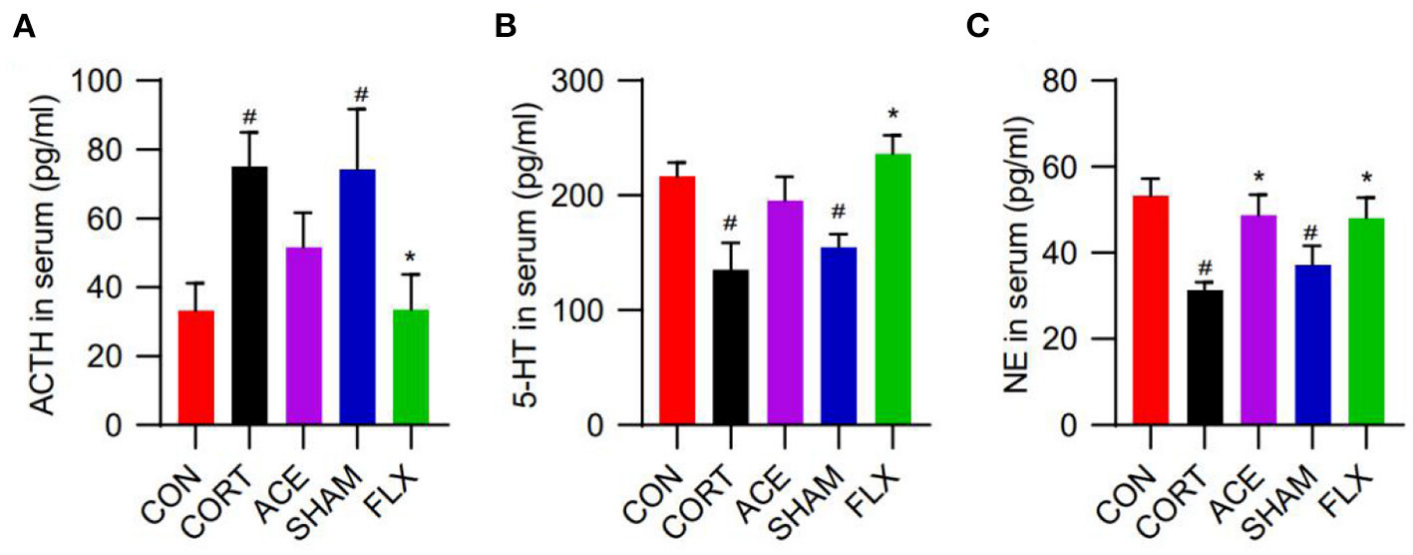
Effect of ACE on the serum neurotransmitter levels of mice. **(A)** ACE decreased the serum ACTH level of mice to some degree without statistical significance (*n* = 8 per group). **(B)** ACE increased the serum 5-HT level of mice to some degree without statistical significance (*n* = 8 per group). **(C)** ACE significantly increased the serum NE level of mice (*n* = 8 per group). Experimental values were expressed as means ± SD. ^#^*P* < 0.05 compared with the CON group. *P < 0.05 compared with the CORT group.

In this work, compared with the control, CORT administration significantly increased the serum ACTH levels of mice, while ACE and FLX treatment reduced the serum level of ACTH (Figure 3A). Moreover, the administration of CORT remarkably decreased the serum levels of 5-HT and NE. Nevertheless, ACE treatment increased the serum level of 5-HT to some degree (Figure 3B). Furthermore, ACE and FLX treatment significantly enhanced the serum levels of NE in the depression mice. Nevertheless, sham-ACE treatment showed no significant effect (Figure 3C).

### Metabolomics Analysis of Brain Tissues

The metabolic profiles of brain samples from the CON, CORT and ACE groups were characterized by LC-MS/MS. The analysis detected a total of 1,684 ion compounds.

### PLS-DA Analysis

**Figure 4:**
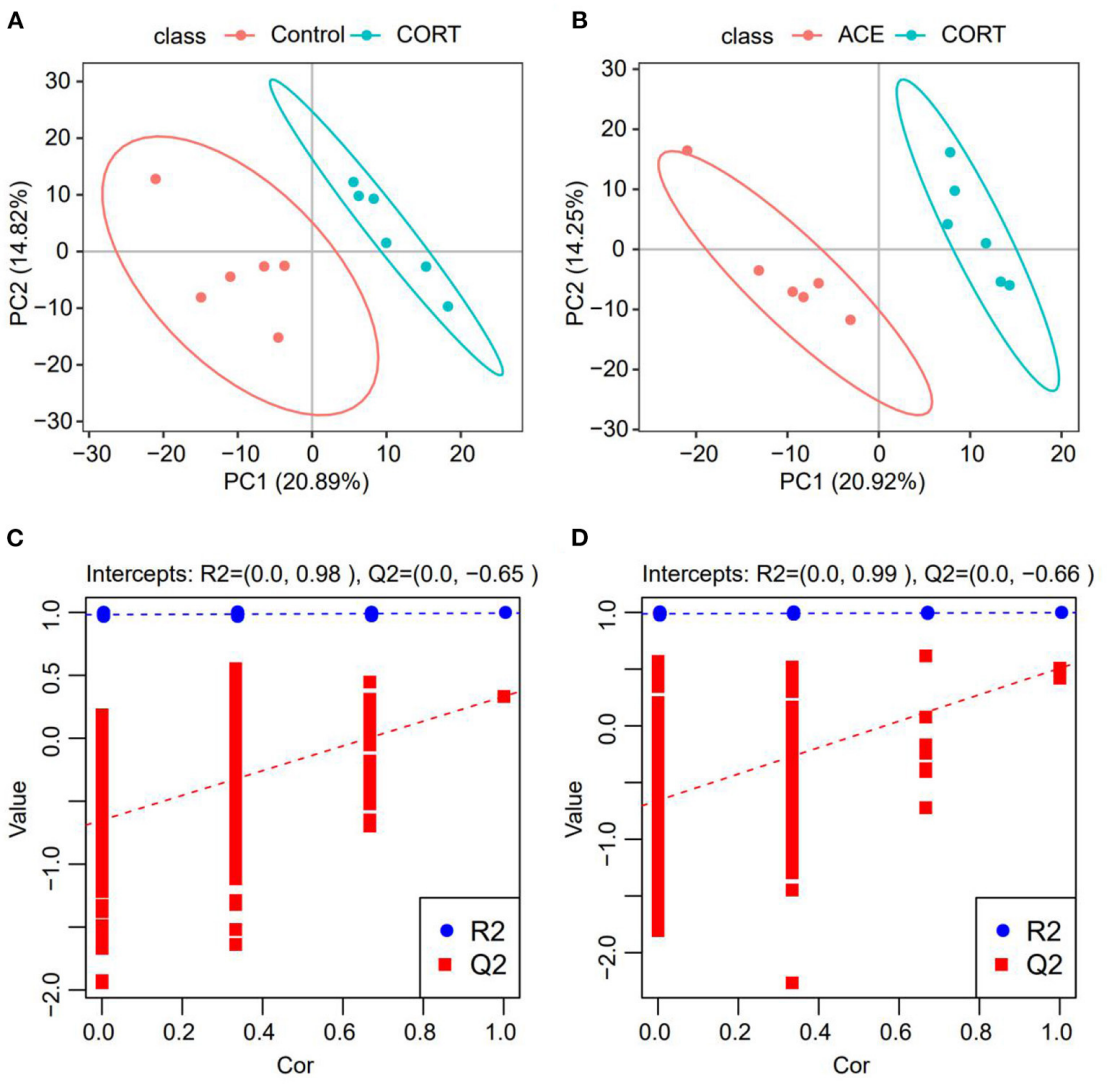
PLS-DA analysis of the samples from the CON, CORT and ACE groups. **(A,B)** Score graph of the PLS-DA analysis model. The horizontal axis is the first principal component, and the vertical axis is the second principal component. The number in parentheses is the score of the principal component, which represents the percentage of the explanation on overall variance of the specific pricipal component. (*n* = 6 per group) **(C,D)** PLS-DA analysis model response permutation testing graph. The two rightmost points in the figure are the actual R2Y and Q2 values of the model, and the remaining points are the R2Y and Q2 values obtained by randomly arranging the samples used. This result is mainly used to judge whether the model is overfit and the validity of the model (*n* = 6 per group).

To obtain the detailed metabolic differences between the CON, CORT and ACE groups, the data were subjected to PLS-DA analysis. Significant differences between the CON and CORT groups were observed in PLS-DA score plot, indicating that the metabolic profiles were dramatically altered following CORT administration. In contrast, the remarkable differences between the CORT and ACE groups in PLS-DA score plot suggested that ACE treatment lead to a significant callback effect on metabolic profiles (Figures 4A,B). The PLS-DA model was validated using the response of the permutation test through 200 permutations, in which all R2 were close to 1 and Q2 were lower than 0. The good PLS-DA model indicated an excellent predictive power (Figures 4C,D).

### Differential Metabolites Screening

**Figure 5:**
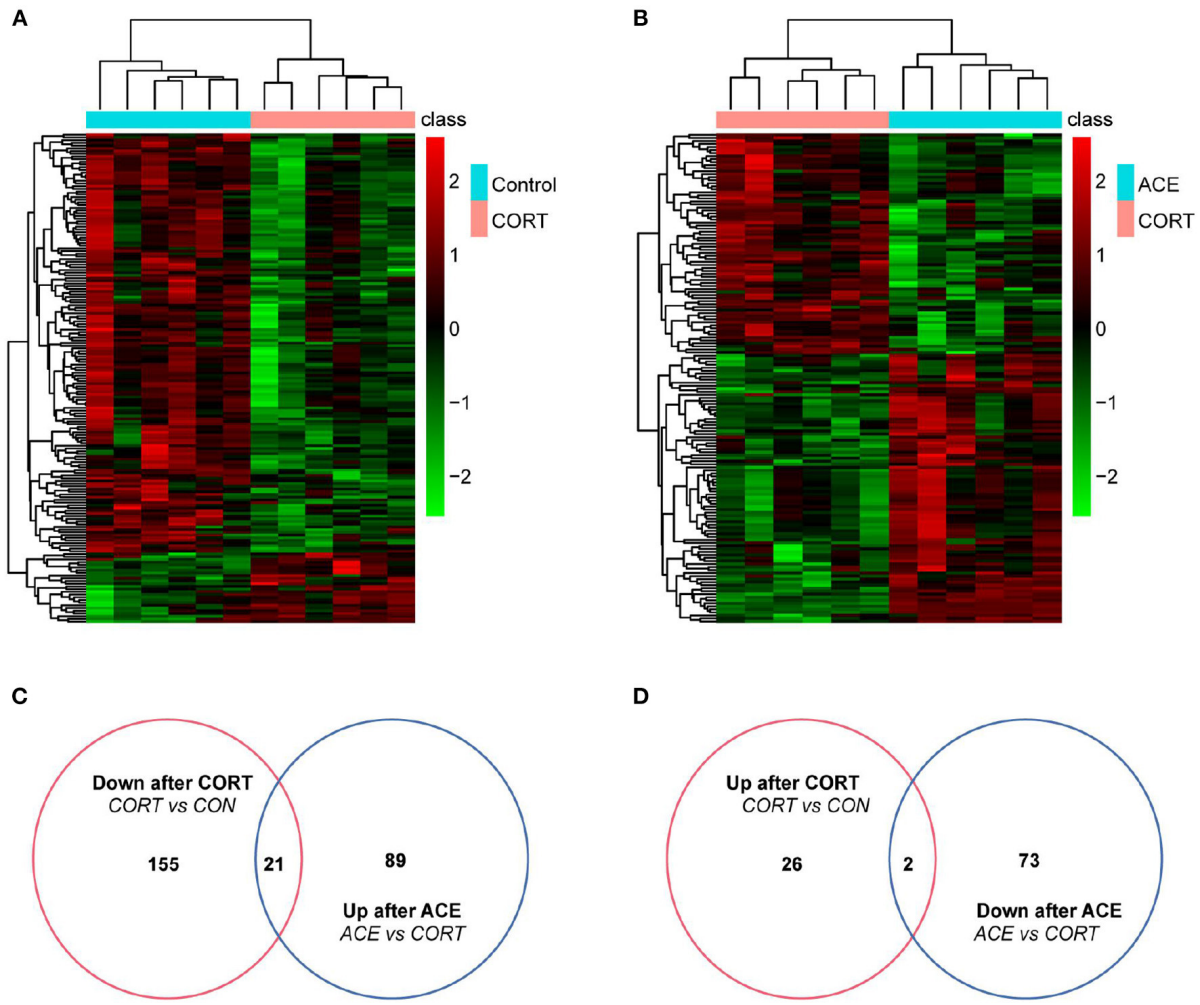
Differential metabolites expression in the brain of mice between the CON, CORT, and ACE groups. **(A)** Heatmap of differentially expressed metabolites between CON and CORT samples with green and red spectrum colors indicating downregulated and upregulated expression, respectively (*p* < 0.05, *n* = 6 per group). **(B)** Heatmap of differentially expressed metabolites between ACE and CORT samples with green and red spectrum colors indicating downregulated and upregulated expression, respectively (*p* < 0.05, *n* = 6 per group). **(C,D)** Venn diagrams showed the overlaps of differentially expressed metabolites between experimental groups. A total of 21 metabolites decreased in CORT group but increased in ACE group. A total of 2 metabolites increased expression in CORT group but decreased in ACE group.

In our present work, the differential metabolites screening conditions were set as following: The VIP of two principal components for the PLS-DA model were ≥ 1. Fold-Change ≥1.2 or ≤ 0.83. *P*-value < 0.05 (Table 1). The results showed that a total of 181 metabolites were significantly and differently expressed in CORT mice compared with the control, with 155 down-regulated metabolites and 26 up-regulated metabolites (Figure 5A). A total of 162 metabolites in the ACE mice relative to the CORT mice displayed significantly differential expression, with 89 metabolites up-regulated and 73 metabolites down-regulated (Figure 5B). ACE treatment reversed 21 down-regulated metabolites induced by CORT administration (Figure 5C). Additionally, ACE treatment reversed two up-regulated metabolites (glycerin, spermidine) induced by CORT administration (Figure 5D). These findings suggested that the behaviors of depression in the mice were significantly attenuated *via* the intervention of ACE, which might be achieved through regulating the above differential metabolites in brain. These differential metabolites may be the treatment targets for ACE to exert its anti-depressive effects.

**Table 1:** A total of 23 differential metabolites in brain were significantly regulated by ACE treatment for its protective effect (VIP ≥ 1, Fold-Change ≥1.2 or ≤ 0.83, *p*-value < 0.05).

**Name**	**Structural formula**	**Value**	**Fold-change**	**variable importance in projection**
Atagabalin	C10H19NO2	0.006015343	2.030789853	2.437886173
NA	C10H22N2O4	0.044708202	2.939875336	2.373943613
To0127900	C9H17NO	0.001059841	1.626339433	2.361939214
NA	C18H31NO2	0.002237832	1.705992477	2.237393434
Glycine	C8H15NO7	0.001126475	1.616287438	2.029389852
Butyryl-l-homosErine lactone	C8H13NO3	0.043026322	1.610630149	1.895197878
Linoleamide	C18H33NO	0.000921998	1.477077597	1.885952314
NA	C15H19 NO5	0.025102575	1.588355158	1.878871577
Tyrosine	C12H16N2O4	0.042120026	1.678155724	1.862066732
Leucine	C6 H13NO2	0.047073181	1.582969519	1.84846739
Hymexazol o-glucoside	C10H15NO7	0.032752292	1.595695617	1.756172084
NA	C9H7N5	0.034528607	1.538238734	1.733662313
(+)-castanospermine	C8H15NO4	0.029853408	1.482080518	1.687325726
Spiroxamine	C18 H35NO2	0.002198635	1.300628861	1.397939511
Valine	C10H20 N2O3	0.012587926	1.474244747	1.387117836
N-acetyl-l-2-aminoadipic acid	C8H13NO5	0.040711557	1.361018957	1.359001501
NA	C9H15NO5	0.011348663	1.367427359	1.345577446
Retinol	C20H30O	0.021802618	1.223678168	1.049871149
2-[(5z,8z,11z)-icosatrienoyl]-sn-glycero-3-phosphoethanolamine	C25H46NO7P	0.026806339	2.77159524	1.01498837
Testosterone	C19H28O2	0.019892157	5.437394848	3.669634374
Glycerin	C23H38O4	0.014765512	0.763749521	2.381849619
Spermidine	C9H21N3O	0.025804694	0.795712175	3.65936226
Cysteine	C3H7NO2S	0.072097044	1.702991174	2.279302811

### Metabolic Pathway Analysis

**Figure 6:**
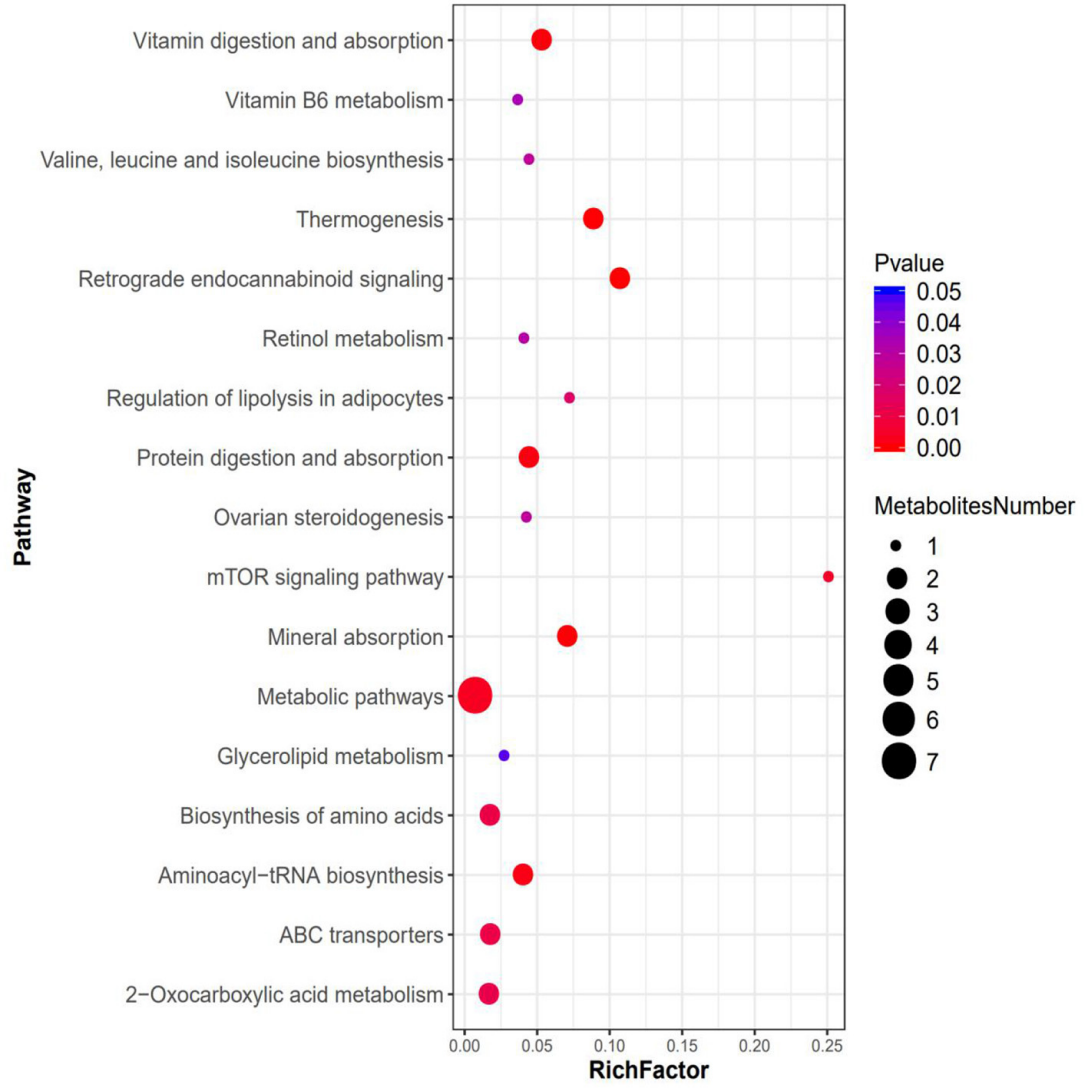
Bubble plots for metabolic pathway enrichment analysis between the ACE and CORT group (*n* = 6 per group). X-axis enrichment factor (RichFactor) is the number of differential metabolites annotated to the pathway divided by all identified metabolites annotated to the pathway. The larger the value, the greater the proportion of differential metabolites annotated to the pathway. The dot size represents the number of differential metabolites annotated to this pathway.

The results of KEGG enrichment analysis showed that metabolic pathway was significantly changed after ACE treatment. There are seven differential metabolites located in metabolic pathway. Furthermore, the regulation of leucine, valine and methionine affect the biosynthesis of amino acids, 2-Oxocarboxylic acid metabolism, mineral absorption and protein digestion, and absorption. In addition, the up-regulation of leucine after ACE treatment regulated the mTOR signaling pathway. Moreover, ACE treatment significantly regulated retrograde endocannabinoid signaling in the brain tissues of depression mice. Additionally, ABC transporters, Aminoacyl-tRNA biosynthesis and other energy signaling pathways might be also involved in the beneficial effects of ACE against depression (Figure 6).

## Discussion

In this study, we established a depression mice model as previously described through CORT repeated administration (28). Chronic CORT administration can mimic the chronic stress associated with the dysfunction of HPA axis in rodents (35). Consistent with previous studies, in this research, the CORT-induced depression mice showed behavioral and neurochemical alterations, which are the characteristics of depression.

Traditional Chinese medicine (TCM) believes that GV20 and GV14 both belong to the Governor Vessel, may function to collect the energy from peripheral regions and transport it to the brain, thus, energizing the entire body (36). GV20 is strongly associated with the brain, and acupuncture at GV20 can regulate cerebral functions (37). GV14 is the convergence of the Governor Vessel with all *yang* meridians (38). According to the theory of TCM, the synergistic and complementary effects of different acupoints compatibility supposedly have an advantage in clinical practice to enhance the curative efficacy and reduce the side effects (39). In clinical practice, GV20 and GV14 are usually used in combination with each other (40). Previous studies demonstrated that GV20 and GV14 are optimized acupoints combinations for neuropsychiatric disorders, which could improve the cerebral blood circulation, enhance memory, and improve antidepressant activity, playing a key role in brain protection (41, 42). Importantly, a clinical study reported that ACE treatment on GV20 and GV14 can effectively regulate the anxiety and depressive symptoms of patients with gastroesophageal reflux disease (43). So, in our work, we investigated the efficacy of ACE treatment at both GV20 and GV14 acupoints on depression model mice. We found that ACE treatment effectively revised the depressive-like behavior in the mice, suggesting that the characteristic presentations of depression could be reversed by ACE treatment. Notably, CORT administration lead to the dysfunction of HPA axis as evident from the marked increase in the serum level of ACTH. However, ACE treatment effectively mitigated the dysfunction of HPA axis as indicated by the reduction in serum ACTH level. Furthermore, ACE treatment reversed the decrease of serum 5-HT and NE levels caused by CORT administration. These results strongly indicated that ACE could improve depressive-like behaviors and regulate neurotransmitter release in depression model mice.

To further determine the mechanism of ACE on depression, we found 23 differential metabolites regulated by ACE treatment in the brain for its positive effect. It is well-known that the neurotransmitters are intimately associated with depression (44). Amino acids are the precursors of these neurotransmitters and depleted levels of them are observed in depression patients (45). Moreover, amino acids metabolism has the potential for predicting the therapeutic response in depression. Importantly, amino acids supplements could alleviate the symptoms of depression (46). Leucine, valine and tyrosine are necessary precursors for the synthesis neurotransmitters. Leucine plays a pivotal role in a variety of physiological and pathological processes, including neuronal function, immunity and aging (47, 48). An integrated meta-analysis demonstrated that the level of leucine was significantly reduced in patients with major depressive disorder (16). Moreover, leucine and valine participate in the formation of 5-HT (49). The low concentration of 5-HT in the synaptic cleft has been implicated as a main etiology of depression (50). Consistent with previous studies, in our present work, we found that the levels of leucine and valine were decreased in the brain tissues of the CORT group, while ACE treatment significantly reversed these effects. Moreover, tyrosine, as an aromatic amino acid, is a precursor for the synthesis of catecholamine neurotransmitters (51, 52). It was reported that tyrosine was essential in neurotransmitters synthesis under the catalysis of tyrosine hydroxylase (53, 54). It should be noted that the pathogenesis of depression is associated with catecholamine neurotransmitters synthesis disturbances, such as dopamine and NE (55, 56). In this work, ACE treatment effectively attenuated the CORT-induced tyrosine reduction in the brain tissues.

Glycine is a type of protective agent, which has been reported to have antioxidant and anti-inflammatory activities *in vivo* (57). Previous works demonstrated that glycine could suppress oxidative stress, neuroinflammation, and apoptotic neurodegeneration in postnatal rat brain (47, 58). Cysteine possesses both anti-oxidant and anti-inflammatory properties (59). Importantly, glutathione, as a tripeptide that functions as an antioxidant, which were formed from cysteine and glycine (60). In this study, ACE significantly enhanced the cysteine and glycine levels in mice with depression, which indicated its neuroprotective efficacy through antioxidant and anti-inflammation mechanisms.

In addition, ACE decreased the levels of glycerin and spermidine in the brain tissues of depression model mice. A large-scale meta-analysis showed that the disorder of lipid metabolism is strongly associated with depression (13). In agreement with previous works, we found that the amount of glycerin was remarkably increased in depression mice. In contrast, ACE effectively reduced the glycerin level (61, 62). Spermidine could promote autophagic activation, which may induce neuronal damage in the hippocampus (61, 62). ACE administration effectively suppressed the level of spermidine in the brain. Additionally, it was found that ACE significantly enhanced the contents of testosterone in the brain. Resent study demonstrated that testosterone could prevent depression through MAPK/ERK2 signaling pathway (63). Linoleamide is an important active ingredient of Maca, which could regulate the energy metabolism and increased the antioxidant capacity (64).

Antioxidant vitamins play an important role in the physiological processes including neuroprotection, oxidative free radical production and immune-modulatory functions. Clinical studies revealed that the serum levels of vitamin A, E, and C were significantly decreased in patients with depression (65). The pathophysiological processes of depression are closely related to oxidative stress and many studies reported that some neurological diseases could be prevented by antioxidant vitamins treatments (66). In this work, it was found that ACE treatment inhibited CORT-induced decrease of retinol (vitamin A). These findings suggested that ACE might exert its therapeutic effects on depression through its antioxidant activities. Taken together, these results suggested that ACE treatment had a notably modulatory effect on the dysregulated cerebral metabolism in the mice with depression. To date, no studies have addressed the alterations of tagabalin, butyryl-l-homos erine lactone, hymexazol o-glucoside, castanospermine, and spiroxamine during depression. These metabolites might has potential in diagnosis and treatment of depression. Herein our results provide evidence on perturbations in the cerebral metabolites of depression.

The results of KEGG enrichment analysis showed that metabolic pathway was significantly changed after ACE treatment. There are seven differential metabolites located in metabolic pathway including glycerin, leucine, valine, pyridoxamine, methionine, testosterone, and retinol. These findings strongly suggested that ACE treatment could regulate amino acid metabolism, lipid metabolism, and vitamin metabolism in depression. Furthermore, the regulation of leucine, valine, and methionine also affect the biosynthesis of amino acids, 2-Oxocarboxylic acid metabolism, mineral absorption, and protein digestion and absorption. In addition, it was suggested that leucine could activate mTOR signaling pathway, which has a protective role in synaptic plasticity in stress and depression (67, 68). In this study, KEGG results revealed that the up-regulation of leucine after ACE treatment regulated the mTOR signaling pathway in depression mice. Moreover, endocannabinoids could regulate mood, emotion, appetite, pain, and cognition *via* the stimulation of cannabinoid receptors. And depression is associated with the decrease in retrograde endocannabinoid signaling in the hippocampus (69). In this work, it was found that ACE treatment significantly regulated retrograde endocannabinoid signaling in the brain tissues of depression mice. Additionally, KEGG analysis demonstrated that ABC transporters, Aminoacyl-tRNA biosynthesis and other energy signaling pathways might be also involved in the beneficial effects of ACE against depression. These results suggest that the integrated regulation of ACE on multipathways and multitargets.

## Conclusion

In conclusion, our study revealed that ACE treatment significantly attenuated the depressive-like behaviors and regulated neurotransmitter release in CORT-induced depression model mice. Importantly, the metabolomics analysis showed that 23 differential metabolites in brain were regulated by ACE treatment for its protective effect. These findings strongly suggested that ACE treatment could ameliorate depression-related manifestations through the attenuation of metabolic dysfunction in brain.

## Data Availability Statement

The original contributions presented in the study are included in the article/supplementary material, further inquiries can be directed to the corresponding author/s.

## Ethics Statement

The animal study was reviewed and approved by the Laboratory Animal Ethics Committee of Guangzhou University of Chinese Medicine.

## Author Contributions

LD and WQ designed and conducted the study with equal contribution. GB, YQ, and SS contributed to the implementation of the experiments. P-CL and YL contributed to the formal analysis and data curation. GX revised the manuscript. QW, ML, and YM supervised the study. All authors contributed to the article and approved the submitted version.

## Conflict of Interest

The authors declare that the research was conducted in the absence of any commercial or financial relationships that could be construed as a potential conflict of interest.

## Publisher's Note

All claims expressed in this article are solely those of the authors and do not necessarily represent those of their affiliated organizations, or those of the publisher, the editors and the reviewers. Any product that may be evaluated in this article, or claim that may be made by its manufacturer, is not guaranteed or endorsed by the publisher.
